# S165. ALTERED ASSOCIATION BETWEEN PARIETAL GRAY-MATTER VOLUME AND DISSOCIATIVE SYMPTOMS IN SCHIZOPHRENIA: A VOXEL-BASED MORPHOMETRY STUDY

**DOI:** 10.1093/schbul/sby018.952

**Published:** 2018-04-01

**Authors:** Huai-Hsuan Tseng, Chui-de Chiu, Kao Chin Chen, I Hui Lee, Po See Chen, Yen Kuang Yang

**Affiliations:** 1 National Cheng Kung University; 2 The Chinese University of Hong Kong

## Abstract

**Background:**

The presence of dissociative symptoms has been constantly reported in patients with schizophrenia. Dissociative-like experience is also part of the prodromal symptoms in those who have higher risk for psychosis. While the underlying neurobiological causes of dissociative symptoms in patients with schizophrenia remains unclear, a history of trauma seems to be related to their dissociative symptoms, as is seen in dissociative disorders. The traumatic experience has been linked to volumetric alterations in patients with schizophrenia. The current study aimed to explore the associations between past traumatic experience, brain volume alteration and the presence of dissociative symptoms in patient with schizophrenia.

**Methods:**

We employed voxel-based morphometry (VBM) to compare the distributions of gray matter volumes (GMV) in 20 patients with schizophrenia (SCZ, 10 Male) and 26 age- and sex-matched healthy volunteers (HV, 11 male). All participants underwent high resolution T1-weighted anatomical images on a 3T MRI system. Past traumatic experience was examined by Brief Betrayal Trauma Survey (BBTS), and the dissociative symptoms were measured by Traumatic Dissociation Scale (TDS).

**Results:**

We found a significant GMV reduction in right thalamus area in SCZ relative to HV group (p=0.01, whole-brain FWE corrected). The GMV in thalamus was negatively associated with high-betrayal traumatic experience in SCZ group (r=-0.48, p=0.033), but not in HV (r=-0.08, p=0.71). While examining the association between GMV and dissociative experience, a significant group by dissociation interaction was observed in the left superior parietal lobule/angular gyrus (SPL/AG) was observed (p=0.024, whole-brain cluster corrected), where negative correlations was observed in HV (r=-0.62, p=0.001) but positive correlations were observed in SCZ group (r=0.67, p=0.001). In SCZ group, both traumatic experience and the left SPL/AG GMV significantly predicted the dissociative experience (p=0.001 and p=0.011, respectively; R2 increased from 0.56 to 0.70 by adding the left SPL/AG GMV into the prediction model).

**Discussion:**

In line with previous literature, we observed a decreased thalamic GMV in SCZ group, which is uniquely associated with their high-betrayal traumatic experience. Superior parietal lobe and angular gyrus has been reported to involved in dissociative experiences in psychiatric illnesses, and modulates sensory, cognitive and self processing in schizophrenia. The positive association we observed between SPL/AG GMV and dissociation experience in SCZ suggests its crucial role in dissociative psychopathology observed in schizophrenia spectrum disorder. Future studies focusing on functional and connectivity alterations of superior parietal/angular area in SCZ and its contribution to dissociative symptoms is warranted.

